# Genetics of prion diseases

**DOI:** 10.1016/j.gde.2013.02.012

**Published:** 2013-06

**Authors:** Sarah E Lloyd, Simon Mead, John Collinge

**Affiliations:** MRC Prion Unit and Department of Neurodegenerative Disease, UCL Institute of Neurology, London, WC1N 3BG, UK

## Abstract

Prion diseases are transmissible, fatal neurodegenerative diseases that include scrapie and bovine spongiform encephalopathy (BSE) in animals and Creutzfeldt–Jakob disease (CJD) in human. The prion protein gene (*PRNP*) is the major genetic determinant of susceptibility, however, several studies now suggest that other genes are also important. Two recent genome wide association studies in human have identified four new loci of interest: *ZBTB38*-*RASA*2 in UK CJD cases and *MTMR7* and *NPAS2* in variant CJD. Complementary studies in mouse have used complex crosses to identify new modifiers such as *Cpne8* and provided supporting evidence for previously implicated genes (*Rarb* and *Stmn2*). Expression profiling has identified new candidates, including *Hspa13*, which reduces incubation time in a transgenic model.

**Current Opinion in Genetics & Development** 2013, **23**:345–351

This review comes from a themed issue on **Molecular and genetic bases of disease**

Edited by **Jim Lupski** and **Nancy Maizels**

For a complete overview see the Issue and the Editorial

Available online 19th March 2013

0959-437X/$ – see front matter, © 2013 Elsevier Ltd. All rights reserved.

http://dx.doi.org/10.1016/j.gde.2013.02.012

## Introduction

Prion diseases or transmissible spongiform encephalopathies are fatal neurodegenerative diseases characterised by long incubation periods, accumulation of abnormal prion protein (PrP^Sc^), spongiosis, gliosis and neuronal loss [1]. They include scrapie and bovine spongiform encephalopathy (BSE) in animals and Creutzfeldt–Jakob disease (CJD) in human. Sporadic CJD typically presents in late middle-old age as a rapidly progressive multifocal cortical dementia with additional neurological features including cerebellar ataxia, pyramidal and extrapyramidal motor dysfunction, myoclonus and dysfunction of the visuoperceptual system. Despite increasing ascertainment, these remain rare conditions, with typical incidences in the developed world of 1–2 cases/million/year. Variant CJD (vCJD), resulting from the human transmission of BSE mainly through dietary exposure, has steadily declined in incidence in the UK since 2000, with a total 176 cases [1,2]. Although the decline in vCJD is most welcome, the prevalence of subclinical infection indicated by anonymous surveys of appendiceal tissue, remains a significant concern at around 1:2000 in the UK (http://www.hpa.org.uk/hpr/archives/2012/news3212.htm#bnrmlprn). Subclinically infected individuals may never convert to clinical cases, however they pose risks for iatrogenic transmission by blood or blood product transfusion, by dentistry and surgery.

PrP is central to the disease process with its misfolded form thought to be the principal component of the infectious particle. Mutations in the prion protein gene (*PRNP*) are causative for inherited prion diseases [3,4] and a common polymorphism (M129V) has a significant effect on susceptibility and phenotype [5,6].

**Figure 1:**
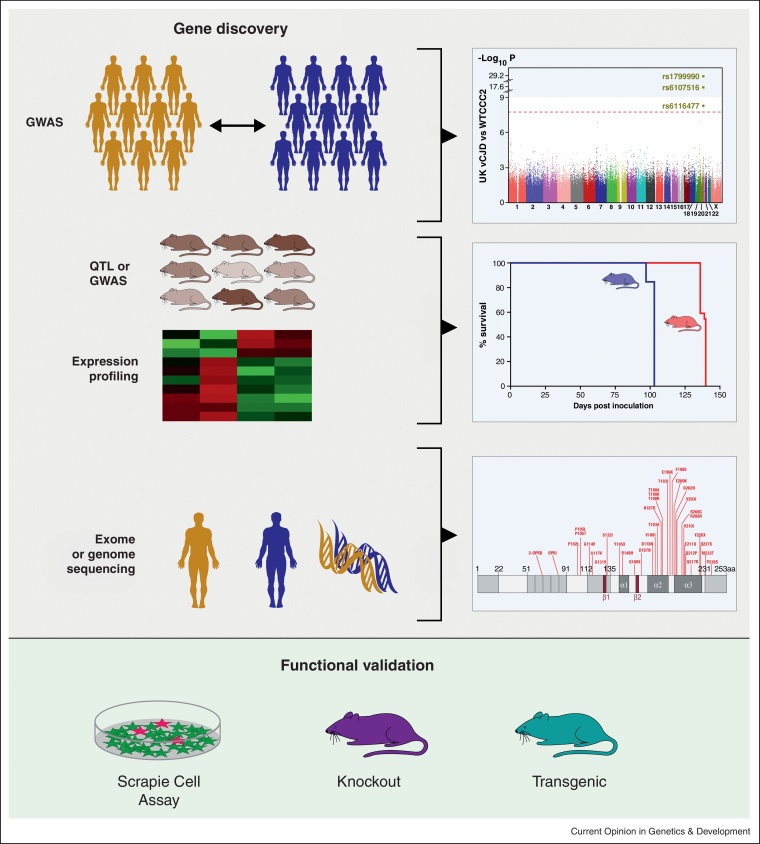
From modifier gene discovery to functional validation. Current strategies for prion disease susceptibility gene discovery include both human and mouse studies. These include human GWAS case–control studies and complementary QTL and GWAS studies in advanced mouse crosses. GWAS results can be displayed as a Manhattan plot as shown here with highly significant hits shown for *PRNP* SNPs. Expression profiling of key tissues for example comparing short (blue) and long (red) incubation time mice has also revealed new pathways and candidates. Next generation sequencing of patients can now be used to identify high risk alleles at novel genes to generate an allelic mutation series as shown here for *PRNP*. Options for functional validation of candidate genes include both *in vitro* (scrapie cell assay) and *in vivo* approaches (mouse models). GWAS – genome-wide association studies; CJD – Creutzfeldt–Jakob disease; QTL – quantitative trait loci; SNP – single nucleotide polymorphism.

In this review we highlight progress since 2010 in determining genetic susceptibility to prion diseases. The use of human genome-wide association studies (GWAS) and complementary mouse studies reinforce the key role of *PRNP* and identify new genetic modifiers. We outline the challenge of verifying the role of putative modifiers and propose a way forward for gene identification and validation (Figure 1).

## Human genetics

Recent work has focussed on the collection of large patient cohorts for GWAS, which has necessarily been an international collaborative endeavour given that human prion diseases are rare. As a generality from common diseases, genetic risk factors discovered by GWAS have been modest in their effects (odds ratios 1–1.2) requiring sample sizes of several thousand to have the statistical power required for unequivocal detection of significant variants. Two collaborative groups are working in prion disease GWAS. The UK MRC Prion Unit in collaboration with the Universities of Munich, Gottingen and Perth has conducted a GWAS of sporadic CJD, variant CJD, iatrogenic CJD, inherited prion disease, and kuru involving over 2000 samples [7,8^••^]. A Europe-wide collaboration led by Dutch and Spanish investigators published a GWAS of vCJD involving 93 samples [9^••^]. In these studies, the *PRNP* locus was unequivocally and strongly associated with risk of prion disease, driven by the known coding variation at *PRNP* codon 129.

In the European vCJD GWAS two single nucleotide polymorphisms (SNPs) (rs4921542 and rs7565981) reached genome-wide significance after pooling discovery and replication populations. Rs4921542 (*p* = 1.6 × 10^−8^) is an intronic variant in the myotubularin related protein 7 gene (*MTMR7*), which is specifically expressed in the central nervous system and dephosphorylates phosphatidylinositol 3-phosphate and inositol 1,3-bisphosphate. Rs7565981 (*p* = 4.2 × 10^−8^) is in an intergenic region upstream of the neuronal PAS (per-ARNT-sim) domain-containing protein 2 gene (*NPAS2*), a regulatory gene belonging to a family of transcription factors.

In the UK-German sporadic CJD study, no non-*PRNP* loci achieved genome-wide signficance. SNPs at the *ZBTB38-RASA2* locus were associated with CJD in the UK (rs295301, *p* = 3.13 × 10^−8^) but these SNPs showed no replication evidence of association in German sporadic CJD or in kuru based tests. Overall, it is likely that the *PRNP* locus contains the only strong risk factors which act universally across human prion diseases. Whilst some genome wide significant loci have been proposed in vCJD, the low incidence of this condition means that there is no way at present to generate unequivocal genetic evidence at these loci. The collective data are most consistent with the findings in other diseases, of strong effects being the exception but many risk loci of modest effects. In prion disease this will require large collaborative GWAS in sCJD to provide definitive statistical evidence of these weak effects.

## The *PRNP* locus and inherited prion disease

**Figure 2:**
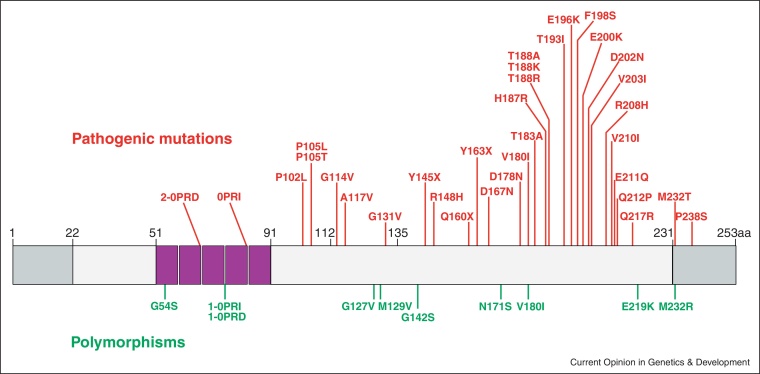
PrP mutations and polymorphisms. A schematic representation of full length human PrP is shown with the cleaved signal sequences shown in grey and the octapeptide repeat region in pink. Disease associated mutations are shown in red and non-synonymous non-pathogenic genetic variants in green. OPRD – octapeptide repeat deletion; OPRI – octapeptide repeat insertion.

Genetic research in human prion disease has not been restricted to GWAS. In inherited prion disease, important information has accrued about which variants are completely penetrant, partially penetrant or simply benign polymorphisms (Figure 2). Several publications originate from groups that routinely sequence *PRNP* and include the distributions of inherited prion disease and new mutations from the UK, China, Japan, US, the Netherlands, and further lessons on how easily inherited prion disease, particularly that caused by truncation mutation, can be mistaken for Alzheimer's disease [10^•^,11–18]. Sequencing the CEPH Human Diversity Panel and the Pakistani population showed that small insertions in the octapeptide repeat region of *PRNP* are probably not pathogenic as they are found in the healthy population, albeit rarely [10^•^,13,19]. Sequencing of the healthy Korean population showed both the M232R and V180I variants implying that these may not be pathogenic mutations [11]. Finally, a study of the rare four octapeptide repeat mutation showed that penetrance of the clinical disease is determined by the genotype at codon 129. When the mutation is linked to codon 129 methionine and the non-mutant allele is also 129 methionine, the disease appears to be penetrant, whereas it is non-penetrant when the non-mutant allele is 129 valine [20].

## Mouse models of prion disease

Human genetic studies provide the most direct link between susceptibility genes and patients, however, these are limited in power and inference regarding mechanisms may be complex. Uniquely amongst neurodegenerative diseases mice are naturally susceptible to prion diseases thus providing an ideal model organism for both gene discovery and hypothesis testing.

Previous mouse quantitative trait loci (QTL) mapping studies using simple crosses have successfully identified many loci linked to prion disease incubation time [21–25]. A new report has added to these data using recombinant inbred lines [26]. Many regions are implicated although only loci on *Mmu11* are replicated between the experimental models. Large regions of this chromosome have also been implicated in previous studies [21–23]. The main disadvantage of these studies is the limited resolution resulting in linkage to very large regions that have proved intractable for candidate gene identification. The availability of advanced crosses such as heterogeneous stocks (HS) of mice and the development of the new Collaborative Cross provide ×10–20 higher resolution and are already providing realistic prospects for identifying individual candidate genes [27–29].

## Heterogeneous stock (HS) mice

The Northport HS was successfully used to fine map and identify candidate genes on *Mmu19* (*Hectd2*) and *Mmu15* (*Cpne8*) [30,31^••^]. For *Mmu15*, the region of linkage was reduced to 3.6Mb from the previous report of 30Mb [24]. Haplotype analysis and genotyping representative SNPs identified *Cpne8* as the most promising candidate. The role of Cpne8 in prion disease has not been established but it may be implicated in PrP processing and targeting as Cpne8 is a member of the copine family that are Ca^2+^ dependent phospholipid binding proteins thought to be involved in membrane trafficking [32].

The identification of *Cpne8* and *Hectd2* highlight the value of HS mice for linkage mapping but they can also be used for association studies, although the existence of large haplotype blocks precludes single gene resolution. This is illustrated by a study to validate two candidates, *RARB* (retinoic acid receptor beta) and *STMN2* (Stathmin-like 2), originally identified as part of a vCJD GWAS [7,31^••^]. Statistical analysis showed a modest association for *Stmn2* but a highly significant association for the *Rarb* locus [31^••^]. Although individual loci have been screened using the HS mice their full potential has not yet been exploited. The advent of high density SNP arrays, similar to those available for the human genome, means that GWAS and copy number variation analysis is now possible. Combined with the availability of genomic sequence for the HS parental strains, this should make candidate gene discovery and validation easier.

## mRNA expression

The use of high density microarrays to look at differential expression of mRNA transcripts during disease progression has identified hundreds of differentially expressed genes and more importantly highlighted gene networks associated with the key cellular processes [33,34]. These studies provide a global view of disease associated changes but are difficult to interpret and many of the pathways may be secondary effects rather than key drivers of the process. We have taken the alternative approach of looking for differential expression between inbred lines of mice with different incubation times. We used uninfected mice and to enrich for relevant genes we looked for a correlation between expression level and incubation time across five lines of mice [35]. Five potential candidates were identified including *Hspa13* (*Stch*), a member of the Hsp70 family of ATPase heat shock proteins. To functionally test *Hspa13* we generated an overexpressing transgenic mouse and following infection with three different prion strains showed highly significant reductions in incubation time. The precise function of Hspa13 is unknown but it has an intra-organellar localisation and is induced by Ca^2+^ release suggesting a role in ER stress and the unfolded protein response (UPR) [36]. It has also been associated with TRAIL-induced apoptosis [37].

## Links to other neurodegenerative diseases

Prion diseases and other neurodegenerative disorders share many common features including familial disease as well as sporadic, aggregation of misfolded protein and neuronal loss. Indeed, there is now evidence that cell to cell spread in these diseases occurs through a ‘prion-like’ mechanism of seeded protein polymerisation [38,39]. The similarities between these diseases had led to causative genes in one disease being tested for an effect in prion disease. Hyperphosphorylated microtubule-associated protein Tau forms the neurofibrillary tangles associated with Alzheimer's disease and frontotemporal dementia and α-synuclein (SNCA) is found in the protein deposits (Lewy bodies) seen in Parkinson's disease (PD). Mice deficient in Tau and SNCA have been challenged with prions and in both cases no difference in incubation time was seen [40,41]. Mutations in *SNCA* are associated with familial PD and in contrast, mice expressing mutant SNCA (A53T) show a reduction in incubation time [42].

## Functional validation

High throughput technologies such as GWAS and expression profiling suggest many candidate genes but the key challenge is to translate this to phenotypic relevance (Table 1). Therefore, the goal is to develop an *in vitro* screen for functional validation. This is being done using neuroblastoma derived cell lines that are highly susceptible to prion infection and are able to sustain chronic infection. The scrapie cell assay (SCA) allows rapid bioassay of prions by counting the numbers of individual infected cells in a culture following serial splits after exposure to an unknown prion isolate and then comparing to standard curves and can be combined with RNAi technology to knockdown gene expression either transiently or stably to investigate the effect if any on prion propagation [35,43]. The assay can be automated and used either in its full format or using chronically infected cells to measure curing of infection when target genes are manipulated. The SCA is prion strain selective and cannot fully substitute for the disease process in brain or the peripheral pathogenesis before neuroinvasion in natural infections and so some important genes will not report in this system. However, the assay should capture genes involved in the fundamentals of cellular prion infection, propagation and clearance thus providing triage for prioritising candidate genes for future studies.

The gold standard for functional validation is to generate a mouse model such as a transgenic, or knockout and look for a perturbation of phenotype such as incubation time. Generating mouse models can be time consuming and expensive, however, rapidly expanding public repositories such as the International Mouse Knockout Consortium (www.knockoutmouse.org) are generating null alleles for all mouse genes in embryonic stem (ES) cell lines which should considerably speed up the process. Alternatives include the use of viral vectors for RNAi delivery to targeted regions of the brain for which proof of concept has already been provided with *Prnp* knockdown [44].

## Conclusions

There is no doubt that genes other than *PRNP* contribute to prion disease susceptibility and considerable progress has been made towards their identification, however, in human it is becoming clearer that there may be many common variants but these are of modest effect. It may be possible to screen larger sets of sporadic CJD, however, the technology is now available for exome or genome sequencing to look for rare variants which may have stronger effects. The mouse genetic data complements the human and the use of GWAS in HS mice promises to deliver more candidate genes. The challenge will be to test these candidates either *in vitro* or *in vivo*. Functionally validated candidates may then be considered as potential therapeutic targets.

## References and recommended reading

Papers of particular interest, published within the period of review, have been highlighted as:

• of special interest
•• of outstanding interest

## Figures and Tables

**Table 1:** Genes implicated in prion disease

Gene or locus	Source (human/mouse)	Comment	Reference
*PRNP*	GWAS (H)	Seen across all human prion diseases and in mouse experimental transmissions.	[7,8^••^,9^••^]

*RARB*	GWAS (H)	vCJD and iCJD	[7,31^••^]
	SNP association (M)	HS mice	

*STMN2*	GWAS (H)	vCJD and kuru	[7,31^••^]
	SNP association (M)	HS mice	

*ZBTB38-RASA2*	GWAS (H)	Sporadic CJD (UK)	[8^••^]
*MTMR7*	GWAS (H)	vCJD	[9^••^]
*NPAS2*	GWAS (H)	vCJD	[9^••^]

*HECTD2*	QTL (M)	vCJD and kuru	[30]
	SNP association (H)	HS mice	

*Cpne8*	QTL (M)	HS mice	[45^•^]
*Stch*	Expression profile and transmission studies (M)	Inbred and transgenic lines	[35]

SNP, single nucleotide polymorphism; vCJD, variant Creutzfeldt–Jakob disease; HS, heterogeneous stock; QTL, quantitative trait locus; GWAS, genome wide association study.
